# Effect of gold nanoparticles distribution radius on photothermal therapy efficacy

**DOI:** 10.1038/s41598-023-39040-6

**Published:** 2023-07-26

**Authors:** Donghyuk Kim, Jeeyong Paik, Hyunjung Kim

**Affiliations:** Department of Mechanical Engineering, Ajou University, Gyeonggi-Do, Suwon-Si, 16499 Korea

**Keywords:** Cancer, Computational biology and bioinformatics, Oncology, Engineering, Nanoscience and technology, Optics and photonics

## Abstract

Lasers are used in various fields, however, in the medical field, they are mainly used for incision or chemotherapy. Photothermal therapy (PTT) is an anti-cancer treatment technique that uses lasers and the photothermal effect to increase the temperature of tumor tissue and induce its death. In this study, the therapeutic effect of PTT using gold nanoparticles as a photothermal converter was analyzed numerically for the occurrence of squamous cell carcinoma inside a skin section consisting four layers. Numerical modeling was implemented to calculate the temperature distribution inside the biological tissue while varying the distribution radius of gold nanoparticles in the tumor tissue, the number of injections, and the intensity of the irradiating laser. For the given situation, the optimal treatment effect was observed when the distribution radius ratio of the injected gold nanoparticles (GNPs) was 1, the number of injections was 7, and the intensity of the irradiated laser was 52 mW. Three apoptotic variables were used to quantitively evaluate the effect of PTT in each case and thus suggest the optimal treatment effect. However, although the temperature range at which apoptosis occurs is known, the maintenance of that temperature range is still under research and the temporal influence of apoptosis remains to be determined.

## Introduction

A laser is a beam of light that has the following characteristics: monochromaticity, coherence, and directivity^1–3^. Lasers are used in various fields, however, in the medical field they are mainly used for incision or chemotherapy^4,5^. Photothermal therapy (PTT), a technique that is mainly used for the treatment of skin cancer, is based on the photothermal effect, a phenomenon in which light energy is converted into thermal energy; it has the advantage of not requiring any incisions, thereby precluding bleeding and secondary infection and enabling fast recovery^6,7^.

PTT uses elevated temperatures to kill tumors^8,9^. Therefore, in PTT research, it is important to prioritize the identification of the mechanisms of temperature-dependent death in biological tissue. In general, temperature-induced tissue death is divided into two main types: apoptosis and necrosis^10,11^. Necrosis involves the leakage of the contents of a tissue as it dies, which, in the case of cancer cells, can result in hazards such as cancer metastasis. Apoptosis refers to a form of tissue self-death, also expressed as “cell suicide”. It is generally accepted that necrosis occurs at cell temperatures above 50 °C, while apoptosis occurs in the range of 43–50 °C. Because of the risk of necrosis, in PTT it is critical to prevent overheating and maintain the temperature of the targeted tissue between 43 and 50 °C, the temperature range in which apoptosis occurs^12–14^.

Regulation of tissue temperature in PTT is done by controlling different laser conditions^15–17^. The optical absorption coefficient of biological tissues is dependent on the wavelength of the incident lasers, with higher optical absorption coefficients at wavelengths in the visible region and lower optical absorption coefficients at wavelengths in the near-infrared region^18,19^. If a laser with a high optical absorption coefficient is utilized, the laser heat will be absorbed not only by the targeted tumor tissue, but by the surrounding normal tissue, increasing the potential for unnecessary thermal damage. As a result, PTT primarily utilizes lasers in the near-infrared region. However, in this region, the low light absorption coefficient of biological tissues prevents the tumor tissue from absorbing much heat. To compensate for this, a photothermal converter is injected into the tumor tissue to increase its coefficient of light absorption. Photothermal converters are materials that enhance the light absorption coefficient at specific wavelengths through the phenomenon of Localized Surface Plasmon Resonance (LSPR) and can be made of various materials such as noble metals and polymers^20–22^. Noble metal photothermal converters are non-toxic and can be effectively excreted from the body after injection, but have a relatively low photothermal conversion efficiency^23,24^. Polymer-based photothermal converters are known to have extremely high photothermal conversion efficiencies depending on the combination of materials, however, there has not yet been an accurate report on their toxicity in the body^25,26^.

Research on PTT is ongoing. Wang et al.^27^ synthesized nanosystems, which combine polyethyleneimine (PEI)-modified gold nanoclusters (AuNCs) with luteinizing hormone-releasing hormone (LHRH) analogues (LHRHa) (AuNCs-LHRHa), to improve the diagnosis and treatment of prostate cancer. Due to their excellent optical/photothermal properties, AuNCs performed well in tomographic imaging and PTT. It was shown that the AuNCs-LHRHa nanosystem was effectively recognized by the gonadotropin-releasing hormone receptor on the RM-1 cell (a murine prostate cancer cell derived from C57BL/6 mice) membrane, which enhanced the tumor cell uptake of the nanosystem, thereby improving the targeting accuracy and PTT effect of prostate cancer. Liu et al.^28^ utilized Fe(III)-doped polyaminopyrrole nanoparticles (FePPyNH_2_ NPs) to suppress tumor recurrence and solve toxicity and side effects after surgical treatment of bladder cancer. The nanoparticles were found to have low cytotoxicity and accumulated in significant amounts in bladder tumors with a blood circulation half-life of 7.59 h. In addition, magnetic resonance imaging (MRI) and photoacoustic imaging (PAI) were used to pinpoint the location of the tumor and, based on the imaging data, the laser was precisely directed at the tumor site. The results confirmed complete resection of the bladder tumor without recurrence with a high photothermal conversion efficiency of 44.3%. Perini et al.^29^ studied the effectiveness of PTT with single and multilayer Ti_3_C_2_T_x_ MXenes (class of two dimensional inorganic compounds composed of nitrides, carbides, or carbonitrides) on two different breast cancer models. PTT was performed in two cancer models using 50 μg/ml of non-toxic MXene, and a significant decrease in tumor survival was observed after treatment along with a significant increase in reactive oxygen species. This confirms that PTT with single and multilayer MXene can regulate tumor growth by expressing apoptosis through temperature elevation. Wang et al.^30^ identified the optimal temperature distribution of the tissue injected with gold nanoparticles (GNPs) to achieve better results in clinical applications. The heat generation in the biological tissue irradiated with the laser was calculated using the Monte Carlo method, and the temperature distribution inside the tissue was calculated using the two-energy equation. In addition, the Arrhenius equation was used to determine the permanent thermal damage of tissues. A parametric study was conducted by varying the intensity of the laser, the volume fraction of GNPs, the heating and cooling time, and the irradiation position, and it was found that controlling the heating and cooling time could effectively prevent overheating of the skin surface and thermal damage to internal tissues. In addition, it was confirmed that lower volume fraction of GNPs achieved better PTT effects when using the ring heating strategy.

As a result, PTT requires a combination of biological, optical, nano, and heat transfer research, and there is still significant scope for improvements. In the field of heat transfer, theoretical research has focused on evaluating the temperature distribution in biological tissue. However, this does not reflect the degree of apoptosis that is commonly used in biology, and simply checks for thermal damage to the tissue through the Arrhenius thermal damage model. In addition, in most of the previous studies, numerical analysis was performed after assuming that the GNPs were uniformly distributed. Furthermore, in the field of medicine and biology, phenomenological studies have only confirmed results under certain predefined conditions and have not determined the optimal conditions for PTT.

Therefore, this study investigated the conditions under which the PTT effect is maximized by changing the distribution radius of GNPs in the tumor tissue. The behavior of the laser particles in the medium was analyzed using the Monte Carlo method and the optical properties of the GNPs were calculated using the Discrete Dipole Approximation method. In addition, the temperature distribution in the medium was calculated based on the heat diffusion equation for a simulated PTT situation. Finally, the effectiveness of PTT was confirmed through the apoptotic variable, which quantitatively judges the degree of maintenance of the apoptosis temperature range during the treatment time and the amount of thermal damage to the surrounding normal tissue, as proposed by Kim et al.^31^ and Kim & Kim^32^, and the optimal treatment conditions were suggested.

## Governing equation and numerical process propagation

### Calculation of light absorption and heat distribution of medium

In this study, the Monte Carlo method was applied to simulate laser irradiation of biological tissue. The Monte Carlo method is a technique for analyzing the behavior of laser particles in a medium whereby the absorption and scattering behavior are analyzed simultaneously to calculate the final light absorption distribution^33^. By calculating the travel distance *S*, deflection angle *θ*, and azimuth angle *ψ* at each time step using random numbers *ξ*, the path of travel in the medium for one particle can be determined. The final distribution of light absorption in the medium can be determined through probability distribution analysis by repeatedly calculating the path for each of the number of particles set initially. The relevant equations are shown below.

1 $$S = \frac{ - \ln (\xi )}{{\mu_{ext} }}$$

2 $$\cos \theta = \left\{ {\begin{array}{*{20}c} {\frac{1}{2g}\left\{ {1 + g^{2} - \left[ {\frac{{1 - g^{2} }}{1 - g + 2g\xi }} \right]^{2} } \right\}{\text{ if }}g{ > 0}} \\ {2\xi - 1{\text{ if }}g{ = 0}} \\ \end{array} } \right.$$

3 $$\psi = 2\pi \xi$$

Equation (1) is the formula for calculating the distance traveled by a given particle in a single time step based on the total attenuation coefficient of the medium (*μ*_*ext*_) and a random number^33^. The total attenuation coefficient can be expressed as the sum of the absorption coefficient (*μ*_*abs*_) and reduced scattering coefficient (*μ*^*’*^_*sca*_). Equations (2) and (3) can be used to calculate *θ* and *ψ*, respectively^33^. For *θ*, there are two applicable different formulae, depending on the value of the anisotropy factor (*g*), which is a dimensionless number indicating the amount of energy retained in the forward direction after a single scattering. Both *θ* and *ψ* are also determined using a randomly selected random number.

In the case of a laser beam, the amount of laser heat absorbed by the medium depends on the profile being irradiated. The profile of the laser is broadly divided into gaussian and top-hat, which can be irradiated into the medium in the form of Eqs. (4) and (5), respectively^33^.

4 $$q(r) = \frac{{2P_{l} }}{{\pi r_{l}^{2} }}\exp \left( { - 2\left( {\frac{r}{{r_{l} }}} \right)^{2} } \right),{\text{ Gaussian}}$$

5 $$q(r) = \left\{ {\begin{array}{*{20}c} {\frac{{P_{l} }}{{\pi r_{l}^{2} }}} & {(if \, r \le r_{l} )} \\ 0 & {(if \, r \ge r_{l} )} \\ \end{array} } \right.{\text{, Top - hat}}$$

Once the distance and angle moved by the particle are calculated, the percentage decrease in energy *W* per time step can be calculated based on the optical properties of the medium, as shown in Eq. (6)^33^. The particle continues to move until, finally, its energy converges to 0. Equations (1–6) are repeatedly calculated for each of the set number of particles. Following that, the final light absorption distribution in the medium can be calculated by calculating the probabilistic particle distribution as shown in Eq. (7), where ${\phi}_{z}$ represents the energy density of the photon in the depth direction and ${\phi}_{rz}$ is the absorbed photon probability density function^33^. In addition, *N* denotes the number of initially selected photons, and ${i}_{r}$ and ${i}_{z}$ are the indices for the grid elements in the *r* and *z* directions, respectively.

6 $$\Delta W = W \cdot \frac{{\mu_{abs} }}{{\mu_{ext} }}$$

7 $$\phi_{z} [i_{z} ] = \sum\limits_{{i_{r} = 0}}^{{N_{r} - 1}} {\phi_{rz} [i_{r} ,i_{z} ] \cdot 2\pi (i_{r} + 0.5)} (\Delta r)^{2}$$

Finally, the light absorption distribution in the medium (Eq. (7)), obtained using the Monte Carlo method, is used in calculation of the temperature distribution in the medium based on the heat diffusion equation, as shown in Eq. (8), and the finite element technique (Eq. (9))^34^.

8 $$\frac{\partial T}{{\partial \tau }} = \frac{{q + \nabla \cdot (k_{m} \nabla T)}}{{\rho c_{v} }}$$

9 $$\begin{gathered} \Delta T = \frac{\Delta \tau }{{\rho c_{v} }}\left( {\mu_{a} FP_{l} + (T_{{x^{ - } }} - T)\frac{{2k_{m} k_{{m,x^{ - } }} }}{{k_{m} + k_{{m,x^{ - } }} }}\frac{1}{{dx^{2} }}} \right. + (T_{{x^{ + } }} - T)\frac{{2k_{m} k_{{m,x^{ + } }} }}{{k_{m} + k_{{m,x^{ + } }} }}\frac{1}{{dx^{2} }} \hfill \\ \, + (T_{{y^{ - } }} - T)\frac{{2k_{m} k_{{m,y^{ - } }} }}{{k_{m} + k_{{m,y^{ - } }} }}\frac{1}{{dy^{2} }} + (T_{{y^{ + } }} - T)\frac{{2k_{m} k_{{m,y^{ + } }} }}{{k_{m} + k_{{m,y^{ + } }} }}\frac{1}{{dy^{2} }} \hfill \\ \, \left. { + (T_{{z^{ - } }} - T)\frac{{2k_{m} k_{{m,z^{ - } }} }}{{k_{m} + k_{{m,z^{ - } }} }}\frac{1}{{dz^{2} }} + (T_{z + } - T)\frac{{2k_{m} k_{{m,z^{ + } }} }}{{k_{m} + k_{{m,z^{ + } }} }}\frac{1}{{dz^{2} }}} \right) \hfill \\ \end{gathered}$$

### Optical properties of gold nanoparticles and biological tissue

The absorption and scattering coefficients in the Monte Carlo method require that the optical properties of nanoparticles and medium must first be calculated. Accordingly, in this study, the discrete dipole approximation (DDA) method was used to calculate the optical properties of GNPs, which were the photothermal converters in this study, and the biological tissues into which the GNPs were injected^35^. This method enables calculation of the optical properties of nanoparticles of various shapes, whereas Mie theory is limited to conventional spherical or elliptical nanoparticles^36^. The DDA method used in this study is detailed below.

First, the polarization vector *P*, which represents the dipole moment in the unit volume, is calculated as shown in Eq. (10) from the polarizability *α* and the local electric field *E*^37^. The variable *E* can be calculated using Eqs. (11) and (12), where *r* and *k* represent the position vector and wavenumber, respectively, and *A* is the interaction matrix between the dipoles, calculated as in Eq. (13) ^37^. If *i* and *j* are equal, the interaction matrix *A* in Eq. (13) can be simplified to *α*_*i*_^*−1*^.

10 $$P_{i} = \alpha_{i} \cdot E_{i} (r_{i} )$$

11 $$E_{i} (r_{i} ) = E_{inc,i} - \sum\limits_{i \ne j}^{N} {A_{ij} \cdot P_{j} (i,j = 1,2,3, \cdots ,N)}$$

12 $$E_{inc,i} = E_{0} e^{{i(k \cdot r_{i} )}}$$

13 $$A_{ij} \cdot P_{j} = \frac{{e^{{i(k \cdot r_{ij} )}} }}{{r_{ij}^{3} }}\left\{ {k^{2} r_{ij} \times (r_{ij} \times P_{j} ) + \frac{{1 - ikr_{ij} }}{{r_{ij}^{2} }} \times \left[ {k^{2} P_{j} - 3r_{ij} (r_{ij} \cdot P_{j} )} \right]} \right\}(i \ne j)$$

The calculated *P* can then be used to calculate the total attenuation *C*_*ext*_, absorption *C*_*abs*_, and scattering cross sections *C*_*sca*_, as shown in Eqs. (14–16), where * indicates the complex conjugate^37^.

14 $$C_{abs} = \frac{4\pi k}{{\left| {E_{0} } \right|^{2} }}\sum\limits_{i = 1}^{N} {\left\{ {{\text{Im}} [P_{i} \cdot (\alpha_{i}^{ - 1} )^{*} P_{i}^{*} ] - \frac{2}{3}k^{3} P_{i} P_{i}^{*} } \right\}}$$

15 $$C_{ext} = \frac{4\pi k}{{\left| {E_{0} } \right|^{2} }}\sum\limits_{i = 1}^{N} {{\text{Im}} (E_{inc,i}^{*} \cdot P_{i} )}$$

16 $$C_{sca} = C_{ext} - C_{abs}$$

This allows the final absorption *Q*_*abs*_, attenuation *Q*_*ext*_, and scattering efficiencies *Q*_*sca*_ of the medium to be calculated as shown in Eq. (17), where *r*_*eff*_ is the effective radius of the nanoparticle (from Eq. (18)) and *V* is its volume^35^.

17 $$Q_{abs} = \frac{{C_{abs} }}{{\pi r_{eff}^{2} }} \, , \, Q_{ext} = \frac{{C_{ext} }}{{\pi r_{eff}^{2} }} \, , \, Q_{sca} = \frac{{C_{sca} }}{{\pi r_{eff}^{2} }}$$

18 $$r_{eff} = \left( {\frac{3V}{{4\pi }}} \right)^{1/3}$$

Once the optical efficiency of a nanoparticle is calculated, the optical properties of the medium, considering the presence of the nanoparticles, can be calculated using the relation proposed by Dombrovsky et al.^38^. The relation is determined by the volume fraction of nanoparticles in the medium *f*_*v*_, their respective optical efficiency, and *r*_*eff*_, and is calculated as in Eqs. (19, 20)^38^. Following the calculation of optical properties of the nanoparticles, the final optical properties of the medium into which the nanoparticles are injected can be calculated as the sum of the calculated optical properties of the nanoparticles and the optical properties of the medium itself, as shown in Eq.(21) ^38^. This makes it possible to calculate the optical properties of the entire medium in the presence of nanoparticles, which can then be applied to the Monte Carlo method.

19 $$\mu_{abs,np} = 0.75f_{v} \frac{{Q_{abs} }}{{r_{eff} }} \, , \, \mu_{sca,np} = 0.75f_{v} \frac{{Q_{sca} }}{{r_{eff} }}$$

20 $$\mu^{\prime}_{sca,np} = \mu_{sca,np} (1 - g)$$

21 $$\mu_{abs} = \mu_{abs,np} + \mu_{abs,m} \, , \, \mu^{\prime}_{sca} = \mu^{\prime}_{sca,np} + \mu^{\prime}_{sca,m}$$

### Verification and comparison of numerical model

**Figure 1:**
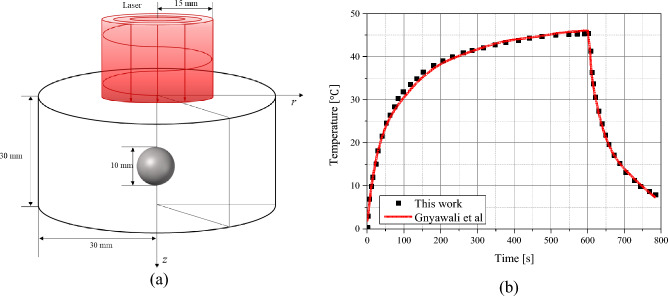
(**a**) schematic of validation model. (**b**) Validation result.

To validate the numerical model presented in this study, a comparative analysis was performed with the results presented by Gnyawali et al.^39^. The study assumed the presence of a spherical tumor with a radius of 5 mm inside a cylindrical biological tissue with a radius of 30 mm and a depth of 30 mm, as shown in Fig. 1a. A laser with a top-hat profile, a wavelength of 805 nm, and a beam radius of 15 mm was utilized, irradiating the top surface with an intensity of 1 W/cm^2^ for 600 s. The initial temperature of the tumor and biological tissues was assumed to be 0 °C. The analysis was performed using the numerical modeling presented in this study under the same conditions as in^39^.

The Fig. 1b shows the results of the numerical simulation verification. The results of^39^ are shown as a red line, and the results of the numerical modeling presented in this study are shown as black scatter points. A root mean square error of 0.6775 was obtained compared to the results of previous studies and, as shown in the graph, the numerical analysis model proposed in this study fits well with previous studies. In addition, a biomimetic phantom was used to validate the numerical model and further demonstrated its validity^32^.

### Numerical geometry and calculation conditions

**Figure 2:**
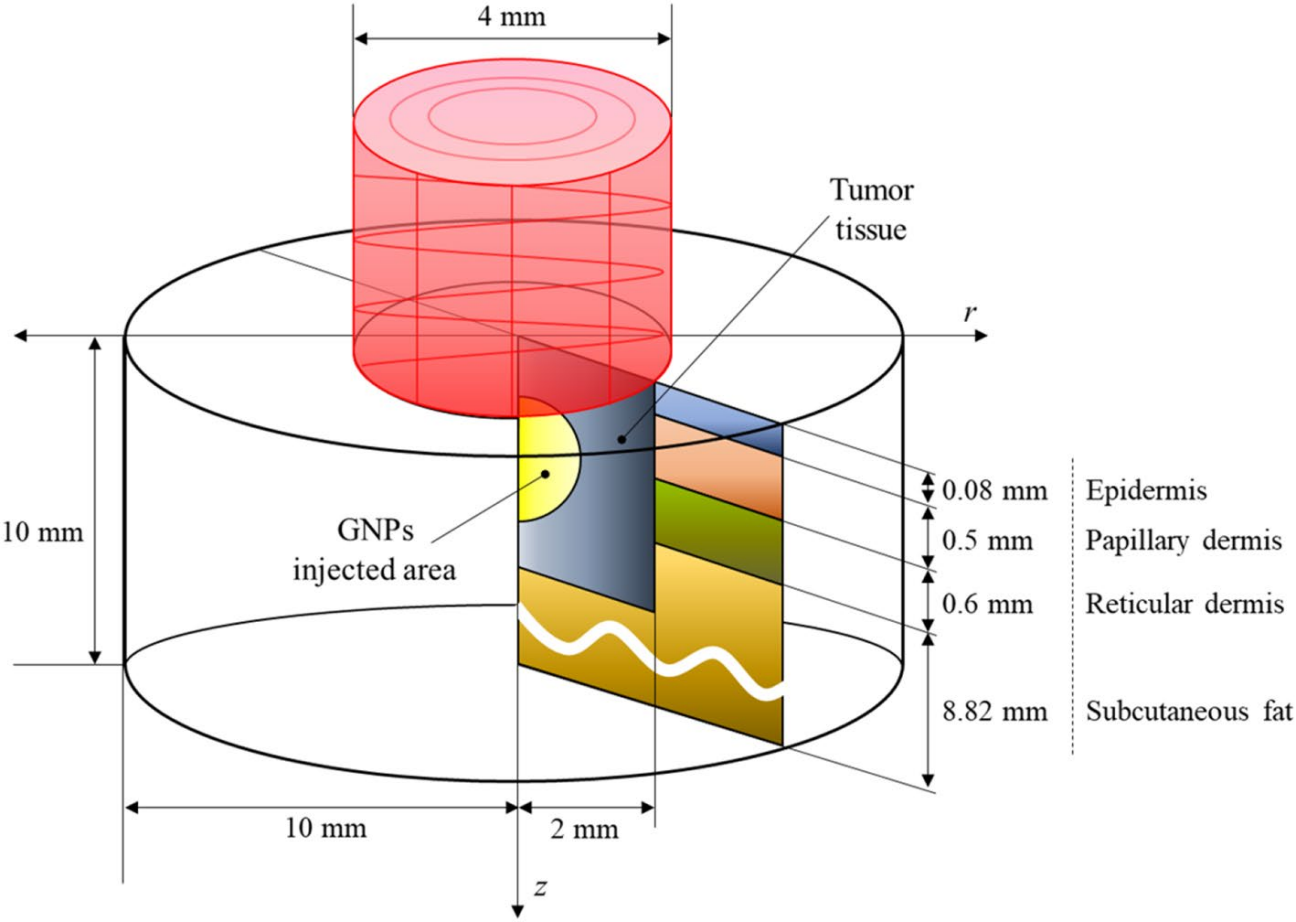
Schematic of numerical model.

A situation in which squamous cell carcinoma (SCC) occurred inside human skin composed of four layers was numerically modeled, as shown in Fig. 2. Assuming that SCC with a diameter of 4 mm and a length of 2 mm occurred in the center of a skin layer with a diameter of 20 mm and a depth of 10 mm, a continuous wave laser with a Gaussian distribution, a wavelength of 1064 nm, and a diameter of 4 mm was irradiated in the perpendicular direction to the skin surface. The laser irradiation time was fixed at 600 s. The length and thermal/optical properties of each skin layer and tumor are shown in Table 1^40–46^.

**Table 1:** Length and thermal/optical properties of tumor tissue and skin layer^40–46^.

	*k*_*m*_ (W/mK)	*c*_*v*_ (J/kgK)	$\rho$(kg/m^3^)	$t$(mm)	${\mu }_{abs}$(1/mm)	${\mu }_{sca}$(1/mm)	*g*
Tumor	0.495	3421	1070	2	0.047	0.883	0.8
Epidermis	0.235	3589	1200	0.08	0.4	45	0.8
Papillary dermis	0.445	3300	1200	0.5	0.38	30	0.9
Reticular dermis	0.445	3300	1200	0.6	0.48	25	0.8
Subcutaneous fat	0.19	2500	1000	8.82	0.43	5	0.75

**Figure 3:**
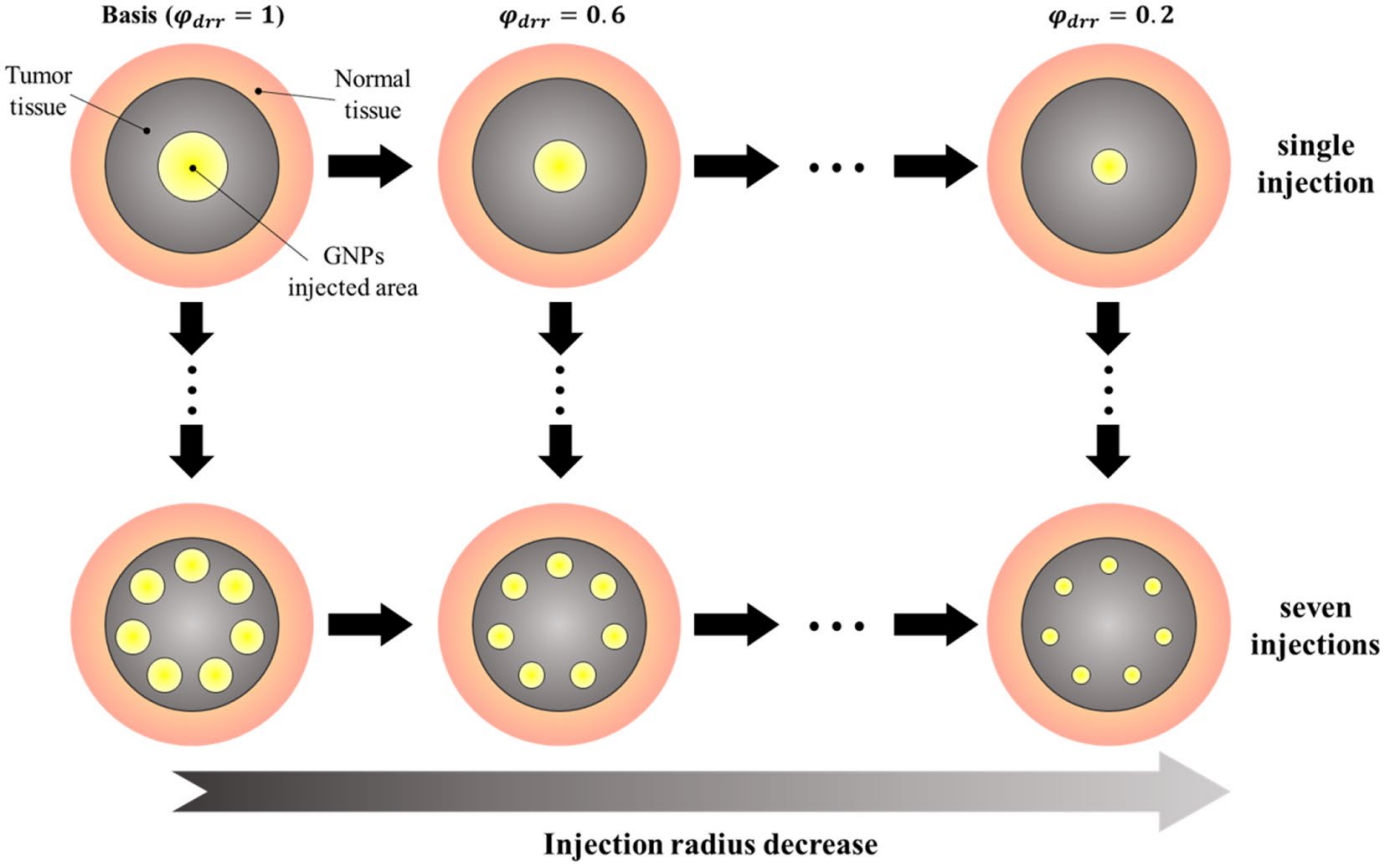
Change the distribution radius of GNPs in the tumor tissue.

In this study, the temperature distribution of the tumor and surrounding normal tissues was investigated while changing the distribution radius of GNPs within the tumor tissue. Figure 3 shows the change in the distribution radius of GNPs in the tumor when viewed in the vertical direction.

Two situations, a single GNPs injection and GNPs deposition over seven injections, are shown as examples. As evident in the figure, the distribution radius of each injection was also varied. Equation (22) shows the distribution radius ratio of GNPs (*φ*_*drr*_), defined as the ratio of the distribution radius of the GNPs in the base case (“Basis”) to the current distribution radius of GNPs.

22 $$\varphi_{drr} = \frac{{r_{dr} }}{{r_{dr,i} }}$$

The total volume of injected GNPs was kept constant between each number of injections and the distribution radius of GNPs was decreased by 20% from the basis over five steps. For the basis, the initial radius of the injected GNPs was 1 mm for a distribution radius ratio of 1 in a single injection; the radius of the injected GNPs decreases as the distribution radius ratio decreases and the number of injections increases. The final numerical solution conditions are summarized in Table 2.

**Table 2:** Parameters of numerical analysis.

Parameter	Case	Number	Remarks
Distribution radius ratio (${\varphi }_{drr}$)	0.2 to 1	5	Interval: 0.2
Number of GNP injections	1 to 7	7	Interval: 1
Laser power (*P*_*l*_)	0 to 100 mW	51	Interval: 2 mW

The distribution radius ratio of GNPs *φ*_*drr*_ was varied from 0.2–1.0 at intervals of 0.2. The number of injections was increased from 1 to 7, at intervals of 1. The distribution radius was changed according to the number of injections. Therefore, there were a total of 35 different cases for the distribution radius of GNPs. Since the total amount of injected GNPs was kept constant between the number of injections, the volume fraction of GNPs in the medium due to each injection decreases as *φ*_*drr*_ decreases and the number of injections increases. The volume fraction of GNPs in the medium was defined as the ratio of the volume of the radius in which the GNPs were distributed to the volume of the entire tumor tissue, from which the optical properties of the space in which the GNPs were distributed were calculated. In addition, numerical analysis was performed by varying the intensity of the irradiated laser from 0–100 mW at 2 mW intervals. Accordingly, the temperature distribution was calculated for 1785 individual cases. Using these results, the PTT conditions that showed the optimal treatment effect were identified.

## Results and discussion

### Result of temperature field

To calculate the apoptotic variable proposed by Kim & Kim^32^ to quantitatively indicate the effectiveness of PTT, it is first necessary to determine the temperature distribution inside the biological tissue. As mentioned above, in this study, the temperature change resulting from varying the distribution radius of GNPs inside the skin cancer tumor was evaluated.

**Figure 4:**
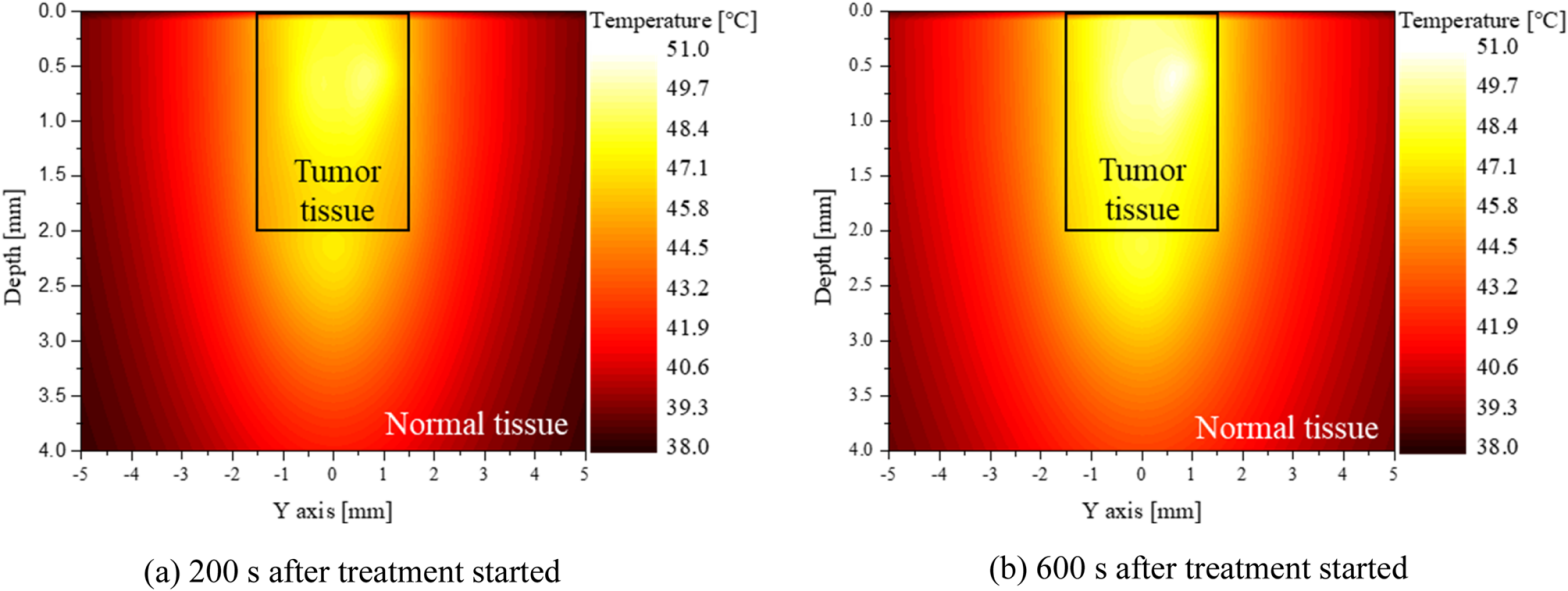
Temperature distribution of medium (*φ*_*drr*_ = 1, number of injected GNPs = 3, *P*_*l*_ = 50 mW).

The Fig. 4 shows the temperature distribution in the YZ plane of normal and tumor tissue at 200 and 600 s after laser irradiation, respectively, for *φ*_*drr*_ = 1, number of injections = 3, and *P*_*l*_ = 50 mW. The laser was set to a wavelength of 1064 nm with a gaussian profile. The area inside the black box represents the tumor tissue. After 600 s had elapsed, the temperature was higher than at the time of 200 s has elapsed. Tumor tissues were confirmed to have entered the apoptosis temperature range for approximately 96% of the tumor volume at 200 s and at 94% at 600 s. However, after 600 s, the normal tissue underlying the tumor tissue reached a temperature of around 48.5 °C, which may cause thermal damage to the normal tissue.

**Figure 5:**
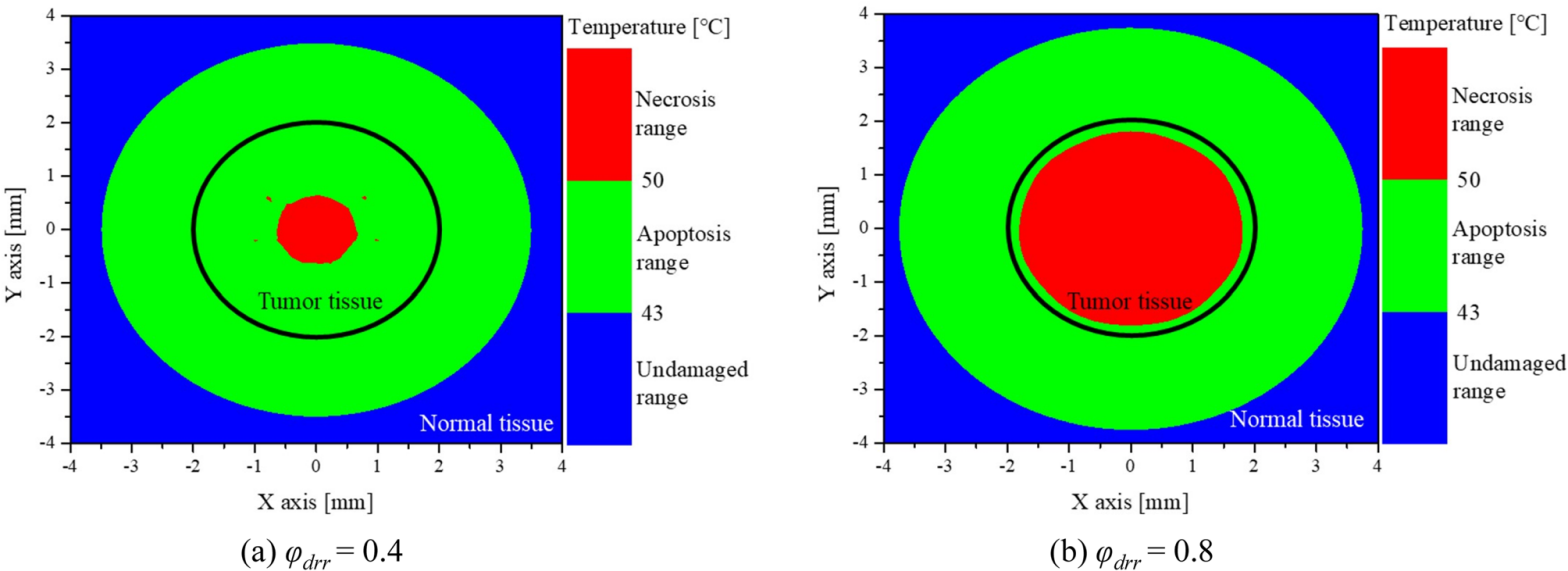
Temperature distribution of medium (depth = 1 mm, number of injected GNPs = 7, *P*_*l*_ = 100 mW, 150 s after treatment started).

The Fig. 5 shows the apoptosis and necrosis areas in the tissue at , depth = 1 mm, number of injections = 7, *P*_*l*_ = 100 mW, and 150 s after the start of treatment. In the figure, the region marked in green represents the apoptosis temperature range, red represents necrosis, and blue represents the region below 43 °C. As shown in the figure, even under the same conditions, the area that satisfies the apoptosis temperature range within the tumor tissue is different depending on *φ*_*drr*_. For *φ*_*drr*_ of 0.4, the percentage of satisfying apoptosis temperature range in tumor tissue was derived to be about 87%, and for *φ*_*drr*_ of 0.8, it was derived to be about 19%. In addition, in the case of normal tissue around the tumor, most of the area is in the apoptosis temperature range, but apoptosis in normal tissue can be considered as thermal damage, so it is necessary to minimize thermal damage by adjusting the appropriate laser intensity.

As illustrated above, this study successfully obtained the temperature distribution inside the medium at each time step in all the considered cases using the proposed method, and quantitatively confirmed the effect of PTT by calculating the apoptotic variable (described in the next section).

### Degree of apoptosis maintenance in the tumor tissue

Using the temperature distribution in the medium calculated in Section "Result of temperature field", the apoptosis retention ratio (${\theta }_{A}^{*}$) was derived to determine the degree of retention of the apoptosis occurrence temperature range inside the tumor tissue. The variable ${\theta }_{A}^{*}$ represents quantitatively how much of the tumor tissue was maintained within the temperature range of 43–50 °C (which, as described earlier, is where apoptosis is known to occur). It is calculated as the ratio of the volume of tumor tissue corresponding to the apoptosis temperature range to the total volume of the tumor tissue (Eq.(23))^32^. Through this variable, it is possible to quantitatively confirm how well apoptotic temperature is maintained during the treatment period.

23 $$\theta_{A}^{*} = \frac{1}{\tau }\int_{0}^{\tau } {\frac{{Apoptosis \, volume \, (if \, 43 < V_{\tau } (T) < 50)}}{Total \, tumor \, volume}} \, d\tau$$

**Figure 6:**
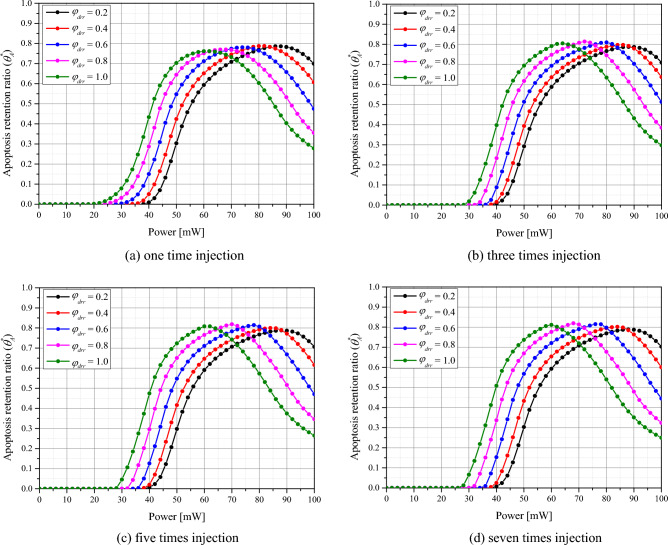
Apoptosis retention ratio (${\theta }_{A}^{*}$) for various number of injected GNPs.

The Fig. 6 plots ${\theta }_{A}^{*}$ as a function of *φ*_*drr*_ and *P*_*l*_ for the number of GNPs injections. The laser was set to a wavelength of 1064 nm with a gaussian profile. First, it was observed that *P*_*l*_ decreased as *φ*_*drr*_ increased for all injections, with ${\theta }_{A}^{*}$ exhibiting a clear maximum in each situation. This is because, as *φ*_*drr*_ increases, the distribution radius of GNPs in the tumor increases, and thus the light absorption area increases, enabling the absorption of more heat and causing a higher temperature rise. Furthermore, a single GNPs injection resulted in a relatively low ${\theta }_{A}^{*}$, which is attributed to the GNPs being concentrated in the central part of the tumor tissue and thus not enabling heating of the entire tumor area. When reviewing all cases, it was found that ${\theta }_{A}^{*}$ was maximized when *φ*_*drr*_ = 0.8, the number of injections = 7, and *P*_*l*_ = 68 mW, indicating that the intratumoral apoptotic temperature is maximized in this case. Compared to the author's previous study, which was analyzed under the assumption that GNPs were uniformly distributed throughout the tumor^47^, it was confirmed that *P*_*l*_ with the maximum ${\theta }_{A}^{*}$ was derived small. In addition, it was confirmed that the optimal value of ${\theta }_{A}^{*}$ itself was derived about 10% higher than the previous result. Compared to the situation in which GNPs are assumed to be distributed throughout the tumor tissue, a relatively small amount of GNPs is injected in the case where GNPs are partially injected, so that the range of absorbing laser heat inside the medium is reduced. Compared to the situation where GNPs are distributed throughout the tumor, the situation where GNPs are partially injected results in a relatively smaller amount of GNPs being injected, leaving less area within the medium to absorb the laser heat. This results in fewer heat-generating zones, which means less unnecessary temperature rise and more efficient temperature control inside the tumor tissue.

However, even if the temperature at which apoptosis occurs in the tumor tissue is maintained for as long as possible, this does not necessarily indicate an optimal treatment condition. the thermal damage to the surrounding normal tissues may also increase further. Hence, evaluation should consider the final treatment effect, including the thermal hazard retention value (as described later).

### Thermal damage to surrounding normal tissues

To reiterate, in PTT, tumor tissue is irradiated with a laser, causing a temperature increase that kills the tumor tissue. The heating itself is concentrated in the tumor tissue, but the temperature of the surrounding normal tissue is also raised through heat conduction between it and the tumor tissue. Excessive temperature increment cause thermal damage to surrounding normal tissue, which needs to be analyzed quantitatively. Therefore, in this study, the thermal hazard retention value (${\theta }_{H}^{*}$) was used to quantify the amount of thermal damage. The variable ${\theta }_{H}^{*}$ is defined as the ratio of the volume of tissue at a temperature indicating thermal damage to the weighted sum of the volume of the surrounding normal tissue; weights are assigned to the various phenomena (e.g., protein denaturation, carbonization) expressed at the various temperature ranges in the biological tissue (Eq.(24))^32^. The surrounding normal tissue was selected to be 50% of the length of the tumor, so the thermal damage in that area can be identified.

24 $$\theta_{H}^{*} = \frac{1}{\tau }\int_{0}^{\tau } {\frac{{\sum\limits_{j = 1}^{m} {V_{n,\tau } (T) \cdot w_{j} } }}{{V_{n,\tau } }}} \, d\tau$$

**Figure 7:**
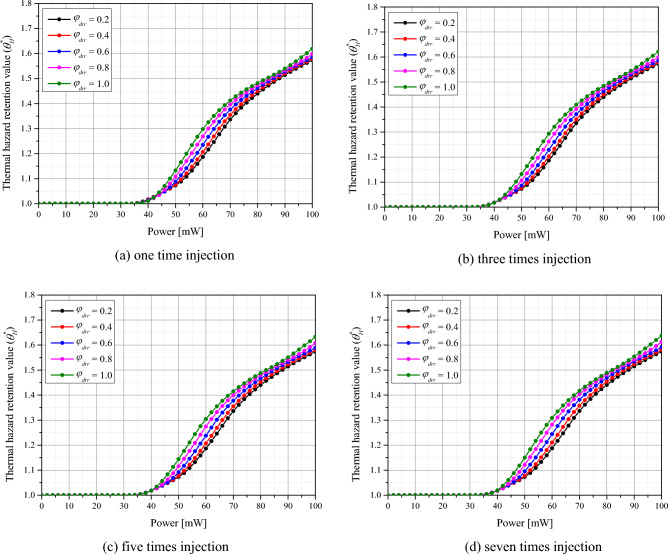
Thermal hazard retention value (${\theta }_{H}^{*}$) for various number of injected GNPs.

The Fig. 7 plots ${\theta }_{H}^{*}$ as a function of *φ*_*drr*_ and *P*_*l*_ for the number of GNPs injections. The laser was set to a wavelength of 1064 nm with a gaussian profile. As expected, as the *P*_*l*_ increases, the values of ${\theta }_{H}^{*}$ increase. It was also observed that, for the same *P*_*l*_* ,* ${\theta }_{H}^{*}$ increased with increasing *φ*_*drr*_, which is explained by the increase in light absorption in the tumor tissue, allowing more heat to be absorbed. Furthermore, it was found that the amount of thermal damage to the surrounding normal tissue was similar regardless of the number of injections. Compared to the situation where GNPs are assumed to be uniformly distributed, it was found that less thermal damage to the surrounding normal tissue was obtained^47^. This is because there are fewer heat-generating areas within the tumor tissue, as mentioned in Section "Thermal damage to surrounding normal tissues", so less heat is conducted to the surrounding normal tissue.

### PTT effect analysis and optimal treatment conditions

Finally, to analyze the effectiveness of PTT, it is necessary to consider both the degree of apoptosis maintenance and the degree of thermal damage to surrounding tissue simultaneously. Since the previously calculated ${\theta }_{A}^{*}$ and ${\theta }_{H}^{*}$ confirm the thermal effects of only tumor tissue and surrounding normal tissue, respectively, the final effect of PTT was analyzed using the effective apoptosis retention ratio ${\theta }_{eff}^{*}$^32^, defined as the ratio of ${\theta }_{A}^{*}$ to ${\theta }_{H}^{*}$. Through this variable, the final PTT effect was quantitatively evaluated to identify the optimal treatment conditions.

**Figure 8:**
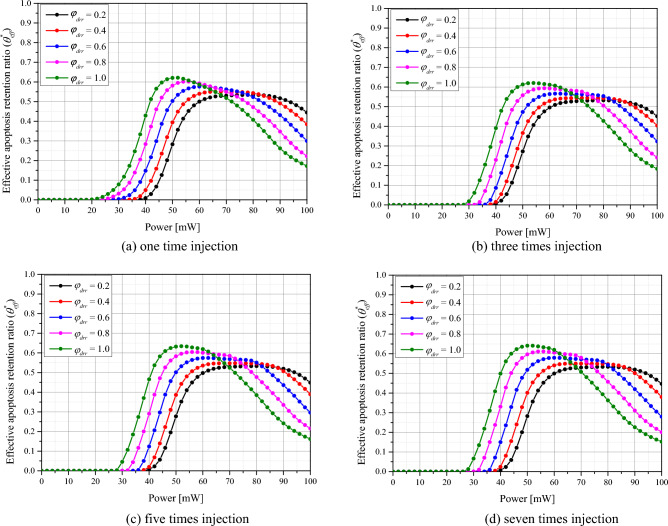
Effective apoptosis retention ratio (${\theta }_{eff}^{*}$) for various number of injected GNPs.

The Fig. 8 plots ${\theta }_{eff}^{*}$ as a function of *φ*_*drr*_ and *P*_*l*_ for various numbers of GNPs injections. The laser was set to a wavelength of 1064 nm with a gaussian profile. A similar trend to that of ${\theta }_{A}^{*}$ was observed with number of injections and it was confirmed that, for a given number of injections and *φ*_*drr*_, there exists a *P*_*l*_ with optimal treatment effect. As *φ*_*drr*_ increased, it was observed that *P*_*l*_ must decrease for ${\theta }_{eff}^{*}$ to have a maximum value. This is because an increase in *φ*_*drr*_ is favorable for maximizing the intratumoral apoptotic temperature, but causes more thermal damage to the surrounding normal tissue. Therefore, it seems that the maximum ${\theta }_{eff}^{*}$ occurs at a lower *P*_*l*_ than the maximum ${\theta }_{A}^{*}$. For a *φ*_*drr*_ of 0.2 and 0.4, the difference in ${\theta }_{eff}^{*}$ with the number of injections was negligible. This is because the distribution radius of GNPs in the tumor tissue is very small, so that an increase in the number of injections makes a negligible difference in the overall ratio. Additionally, similar to the results for ${\theta }_{A}^{*}$, a single injection of GNPs resulted in a relatively low ${\theta }_{eff}^{*}$ compared to greater numbers of injections. This is because, in a single injection, the distribution of GNPs is concentrated in the center of the tumor tissue, hence only the temperature near the center rises to reach or exceed the apoptotic temperature range. After analyzing all cases, it was found that ${\theta }_{eff}^{*}$ is maximized when *φ*_*drr*_ = 1, the number of injections = 7, and *P*_*l*_ = 52 mW. These the treatment conditions produce the optimal therapeutic effect in theory, however, in the actual treatment situation, if the values of ${\theta }_{eff}^{*}$ do not differ significantly, treatment conditions that maximizes ${\theta }_{A}^{*}$ to cause more tumor tissue death may be preferable, even at the expense of thermal damage to the surrounding normal tissue, from the perspective of cancer cell death.

## Conclusions

In this study, the effectiveness of PTT based on the number of GNPs injections and GNPs distribution radius in tumor tissue was quantitatively determined through numerical analysis. Numerical simulation modeling was implemented to calculate the temperature distribution inside the medium for a situation in which SCC was assumed to have developed inside a skin layer consisting of four layers and the tumor tissue, partially injected with GNPs, was irradiated with a laser. The behavior of the laser particles transmitted inside the biological tissue was analyzed using the Monte Carlo method and the optical properties of the used GNPs were calculated by DDA method.

Numerical simulations were performed to determine the temperature distribution in the medium for a given total number of GNPs while varying the number of GNPs injections into the tumor tissue, distribution radius of GNPs, and the intensity of the laser. Three metrics were used to quantitatively determine the therapeutic effect of PTT: the apoptosis retention ratio, a variable that indicates how long the temperature inside the tumor tissue is maintained within 43–50 °C (the temperature range over which apoptosis occurs); the thermal hazard retention value, a variable that quantitatively verifies the amount of thermal damage to the surrounding normal tissue; and the effective apoptosis retention ratio, a variable combining the former two metrics. For the given situation, the optimal treatment effect was observed when the distribution radius ratio of the injected GNPs was 1, the number of injections was 7, and the intensity of the irradiated laser was 52 mW. However, as mentioned in Section "PTT effect analysis and optimal treatment conditions", in the actual treatment, it may be necessary to choose conditions that maximize the expression of apoptosis in the tumor tissue at the expense of thermal damage to the surrounding normal tissue. In addition, since this study was only based on numerical analysis, it is considered necessary to verify it through future experimental studies. Lastly, although the temperature range at which apoptosis occurs is known, the maintenance of that temperature range is still under research and the temporal influence of apoptosis remains to be determined.

### Informed concent

D.K received his Ph.D. in Mechanical Engineering in 2023 from Ajou University, Korea. His research interests are bioheat transfer, photothermal therapy, air conditioning, secondary battery and thermal engineering. J.P is master student in Ajou University, Korea. His research interests are bioheat transfer and house appliance. H.K is a Professor in Department of Mechanical Engineering at Ajou University, Korea. His research interests are nano fluidics, optical property measurement, micro scale heat and mass transfer and measurement with Micro PIV, LIF.

## Data Availability

All data generated or analysed during this study are included in this published article.

## List of symbols

*C*: Cross-sectional area (${\mathrm{m}}^{2}$)
*c*_*v*_: Specific heat ($$\mathrm{J}/\mathrm{kgK}$$)
*E*: Electric field ($$\mathrm{N}/\mathrm{C}$$)
*F*: Fluence rate ($$1/{\mathrm{m}}^{2}\mathrm{s}$$)
*g*: Anisotropy factor
*i*: Index of grid element
*k*: Wavenumber of radiation ($1/\mathrm{m}$)
*k*_*m*_: Thermal conductivity ($$\mathrm{W}/\mathrm{mK}$$)
*N*: Number of photons
*P*: Polarization vector ($$\mathrm{C}/{\mathrm{m}}^{2}$$)
*P*_*l*_: Intensity of laser (W)
*q*: Volumetric heat source ($\mathrm{W}/{\mathrm{m}}^{3}$)
*Q*: Dimensionless efficiency factor
*r*: Position vector
*r*_*eff*_: Effective radius of nanoparticle (m)
*S*: Photon’s moving distance per 1 step (m)
*t*: Thickness (m)
*T*: Temperature (K)
*W*: Energy weight of photon (J)

## Greek symbols

$$\alpha$$: Polarizability ($${\mathrm{C}}^{2}{\mathrm{m}}^{2}/\mathrm{J}$$)
$$\theta$$: Deflection angle (°)
${\theta }_{A}^{*}$: Apoptosis retention ratio
${\theta }_{eff}^{*}$: Effective apoptosis retention ratio
${\theta }_{H}^{*}$: Thermal hazard retention value
$$\mu$$: Optical coefficient (1/m)
${\mu }{\prime}$: Reduced optical coefficient (1/m)
$$\xi$$: Random number
$$\rho$$: Density ($$\mathrm{kg}/{\mathrm{m}}^{3}$$)
${\phi }_{z}$: Energy density of photon
${\phi }_{rz}$: Absorbed photon probability density
$$\tau$$: Time (s)
$$\psi$$: Azimuth ($$^\circ$$)
${\varphi }_{drr}$: Distribution radius ratio of GNPs

## Subscripts

*abs*: Absorption
*dr*: Distribution radius
*ext*: Extinction or total
*m*: Medium
*np*: Nano particle
*sca*: Scattering
*x, y, z*: Notation of direction

## Superscripts

** + **: Next element
−: Previous element
